# Troglitazone Attenuates TGF-β1-Induced EMT in Alveolar Epithelial Cells via a PPARγ-Independent Mechanism

**DOI:** 10.1371/journal.pone.0038827

**Published:** 2012-06-20

**Authors:** Beiyun Zhou, Stephen T. Buckley, Vipul Patel, Yixin Liu, Jiao Luo, Manda Sai Krishnaveni, Mihaela Ivan, Lucas DeMaio, Kwang-Jin Kim, Carsten Ehrhardt, Edward D. Crandall, Zea Borok

**Affiliations:** 1 School of Pharmacy and Pharmaceutical Sciences, Trinity College, Dublin, Ireland; 2 Will Rogers Institute Pulmonary Research Center, Division of Pulmonary, Critical Care and Sleep Medicine, Department of Medicine, University of Southern California, Los Angeles, California, United States of America; 3 Department of Biochemistry and Molecular Biology, University of Southern California, Los Angeles, California, United States of America; Children's Hospital Los Angeles, United States of America

## Abstract

Peroxisome proliferator activated receptor γ (PPARγ) agonists are effective antifibrotic agents in a number of tissues. Effects of these agents on epithelial-mesenchymal transition (EMT) of primary alveolar epithelial cells (AEC) and potential mechanisms underlying effects on EMT have not been well delineated. We examined effects of troglitazone, a synthetic PPARγ agonist, on transforming growth factor (TGF)-β1-induced EMT in primary rat AEC and an alveolar epithelial type II (AT2) cell line (RLE-6TN). TGF-β1 (2.5 ng/mL) induced EMT in both cell types, as evidenced by acquisition of spindle-like morphology, increased expression of the mesenchymal marker α-smooth muscle actin (α-SMA) and downregulation of the tight junctional protein zonula occludens-1 (ZO-1). Concurrent treatment with troglitazone (or rosiglitazone), ameliorated effects of TGF-β1. Furthermore, following stimulation with TGF-β1 for 6 days, troglitazone reversed EMT-related morphological changes and restored both epithelial and mesenchymal markers to control levels. Treatment with GW9662 (an irreversible PPARγ antagonist), or overexpression of a PPARγ dominant negative construct, failed to inhibit these effects of troglitazone in AEC. Troglitazone not only attenuated TGF-β1-induced phosphorylation of Akt and glycogen synthase kinase (GSK)-3β, but also inhibited nuclear translocation of β-catenin, phosphorylation of Smad2 and Smad3 and upregulation of the EMT-associated transcription factor SNAI1. These results demonstrate inhibitory actions of troglitazone on TGF-β1-induced EMT in AEC via a PPARγ-independent mechanism likely through inhibition of β-catenin-dependent signaling downstream of TGF-β1, supporting a role for interactions between TGF-β and Wnt/β-catenin signaling pathways in EMT.

## Introduction

Idiopathic pulmonary fibrosis (IPF) is a progressive disorder of unknown etiology characterized by accumulation of fibroblasts/myofibroblasts and marked deposition of extracellular matrix components [1]. Epithelial-mesenchymal transition (EMT), a process whereby epithelial cells lose their phenotypic characteristics and acquire mesenchymal features, has been suggested as a mechanism that may contribute to fibroproliferation in pulmonary fibrosis [2]–[5]. Currently, there is no effective treatment to improve prognosis for IPF patients [6], [7]. Given the lack of treatment options and the possible contribution of EMT to the pathogenesis of IPF, pharmacologic inhibition of EMT may represent a novel therapeutic approach. Such inhibition could have the effect of slowing or reversing established fibrosis of the lung.

Cumulative evidence, both *in vivo* [5] and *in vitro* [8], indicates that transforming growth factor (TGF)-β1 is a primary regulator of EMT. Development of strategies to inhibit active TGF-β1 and its associated activities appears to be an attractive approach to prevention of EMT and/or IPF. Recent investigations have revealed that ligands of peroxisome proliferator-activated receptor gamma (PPARγ) are capable of opposing profibrotic effects of TGF-β1 [9]–[11]. Additionally, in epithelial cells of the airways, such ligands serve to inhibit proinflammatory cytokine release and contribute to regulation of cellular differentiation [12], further implicating them in the fibrotic process. PPARγ ligands include endogenous agents such as the cyclopentenone prostaglandin 15-deoxy-Δ12,14-prostaglandin J2 (15d-PGJ2) and a group of synthetic compounds known as thiazolidinediones (TZDs) that are currently in clinical use for their anti-diabetic effects. Of note, certain biological actions of TZDs have been shown to occur independently of PPARγ [11], [13].

In murine models, TZDs ameliorate bleomycin-induced lung fibrosis [14]–[16]. Specifically, they have been shown to inhibit TGF-β1-induced differentiation of lung fibroblasts to myofibroblasts [9], [11], [15] as evidenced by suppression of α-smooth muscle actin (α-SMA) upregulation, and effects appear to be mediated via both PPARγ-dependent [9] and -independent mechanisms [9], [11]. In the context of EMT, recent studies in retinal pigment and renal proximal tubule epithelial cells have demonstrated that some PPARγ ligands inhibit EMT induced by either TGF-β1 or high glucose, respectively [10], [17]. In the lung, inhibitory effects of TZDs on EMT have been shown in a lung adenocarcinoma cell line (A549) [18], [19] to be PPARγ-independent. However, conflicting results with regard to Smad-dependence or -independence of inhibitory effects of TZDs emerged from these studies. It is not known if these results and underlying mechanisms can be extrapolated to non-transformed alveolar epithelial cells (AEC).

In the current study, we examined the effects of troglitazone, a synthetic PPARγ ligand, on TGF-β1-mediated EMT in both primary AEC and a non-transformed rat lung epithelial cell line, RLE-6TN [20]. Results reveal that troglitazone attenuates transition of both primary AEC and RLE-6TN cells to myofibroblasts, effects that are independent of PPARγ. Troglitazone inhibited EMT-related phosphorylation of Akt, GSK-3β and Smad2/Smad3, and two key downstream events (β-catenin nuclear translocation and SNAI1 activation), suggesting that effects of troglitazone are mediated by β-catenin-dependent signaling downstream of TGF-β. Given the importance of EMT in IPF, our findings point to a potential therapeutic role for TZDs in this disorder.

### Culture of RLE-6TN Cells

RLE-6TN cells, a rat alveolar epithelial type II (AT2) cell line, were purchased from American Type Culture Collection (Manassas, VA). Cells were maintained in Dulbecco’s Modified Eagle’s medium, nutrient mixture F-12 Ham supplemented with 10% fetal bovine serum, 40 mmol/L HEPES, 100 U/ml penicillin G and 100 µg/ml streptomycin. For EMT studies, cells were allowed to attach overnight in media alone. For the majority of experiments, cells were maintained in either media alone or media supplemented with 2.5 ng/ml TGF-β1 (R&D Systems, Minneapolis, MN) with or without 10 µM troglitazone (Cayman Chemical, Ann Arbor, MI) for 3 days. Dose response effects of troglitazone (or rosiglitazone) were investigated at concentrations from 0 to 20 µM (or from 10-40 µM), respectively. Cultures were maintained in a humidified 5% CO_2_ incubator at 37°C, and all media and additives were replaced every other day, starting on day 2.

### Primary AEC Isolation and Culture

AT2 cells were isolated from adult male Sprague-Dawley rats by elastase disaggregation (2.0–2.5 U/ml) and panning on rat IgG-coated bacteriological plates [21]. All animals were treated in accordance with the guidelines and approval of the University of Southern California Institutional Animal Care and Use Committee. AT2 cells were resuspended in minimal defined serum-free medium (MDSF) [21]. Cells were seeded into 1.1-cm^2^ tissue culture-treated polycarbonate (Nuclepore) filter cups (Transwell; Corning Costar, Cambridge, MA). Media were supplemented with 100 µg/ml *cis*-OH-proline (Sigma, St. Louis, MO) for the first 24 to 48 hours of culture to selectively eliminate fibroblasts [22]. Cells were subsequently maintained in MDSF or in MDSF supplemented with 2.5 ng/ml TGF-ß1 (R&D Systems) with or without 10 µM troglitazone in both apical and basolateral compartments for up to 12 additional days (for a total of 14 days). Equivalent amounts of vehicle for each supplement (4 mM HCl containing 1 mg/ml of bovine serum albumin (BSA) in the case of TGF-ß1 and dimethyl sulfoxide (DMSO) in the case of troglitazone) were added to control cultures. Cultures were maintained in a humidified 5% CO_2_ incubator at 37°C. Media were changed every other day. Cell viability (>95%) was measured by trypan blue dye exclusion. In studies investigating the impact of GW-9662 (Sigma), an irreversible PPARγ antagonist, cells were treated with TGF-β1 (2.5 ng/ml) ± troglitazone (10 µM) ± GW9662 (1.0–7.5 µM).

### Monolayer Transepithelial Electrical Resistance (R_t_ *)*

*R_t_* (KΩ·cm^2^) was measured using a rapid screening device (Millicell-ERS; Millipore, Bedford, MA). Effects of TGF-β1 supplementation (in the presence or absence of troglitazone) on *R_t_* were evaluated on days 3, 5, 7, 9, and 10 following plating.

### Western Analysis

Cells were lysed in 2% sodium dodecylsulfate (SDS) lysis buffer (62.5 mM Tris-HCl, 2% SDS and 10% glycerol) on ice for 30 min and briefly sonicated. Protein sample concentrations were determined using a standard protein concentration assay (Bio-Rad, Hercules, CA). Samples were separated by SDS-polyacrylamide gel electrophoresis and transferred to Immuno-Blot polyvinylidene fluoride membranes (Bio-Rad). Membranes were blocked in 5% nonfat dry milk in Tris-buffered saline with Tween (TBS-T; pH 7.4) for 1 h at room temperature (RT). Incubation with primary antibodies was carried out overnight at 4°C, and with horseradish peroxidase-conjugated secondary antibodies at RT for 1 h. Primary antibodies for α-SMA, FLAG and β-catenin were obtained from Sigma and ZO-1 antibody was purchased from Invitrogen (Carlsbad, CA). Phospho-Akt (Ser473), total Akt, phospho-Smad2, total Smad2, phospho-Smad3, total Smad3, phospho-GSK-3β and total GSK-3β antibodies were purchased from Cell Signaling (Danvers, MA), and all secondary antibodies were obtained from Promega (Madison, WI). Peroxidase activity was detected with Super Signal (Pierce, Rockford, IL) and images analyzed using a FluorChem imager (Alpha Innotech, San Leandro, CA). To ensure equal loading, protein levels were normalized to the levels of lamin A/C, glyceraldehyde 3-phosphate dehydrogenase (GAPDH) or β-actin detected using anti-lamin A/C polyclonal antibody (Santa Cruz Biotechnology, Santa Cruz, CA), anti-GAPDH monoclonal antibody (Abcam, Cambridge, MA) or anti-β-actin monoclonal antibody (Sigma), respectively.

### Production of Lentivirus in 293T Cells

PPARγ dominant negative expression plasmid, LV-PPARγ-DN (human PPAR LV-PPARγ-DN 1-L466A/E469A mutant cloned in pCDH1-MCS1-EF1-copGFP vector) was kindly provided by R.P. Phipps (University of Rochester, Rochester, NY). Infectious lentivirus was created by cotransfection of LV-PPARγ-DN or LV-control (pCDH1-MCS1-EF1-copGFP) with pCMVΔR8.91 and pMD.G into human 293T cells. Virus was harvested after 48 hours, filtered through 0.45 µm filters, concentrated with PEG-it virus precipitation solution (System Biosciences, Mountain View, CA ) and titered with HIV p24 ELISA (Cell Biolabs, San Diego, CA).

**Figure 1:**
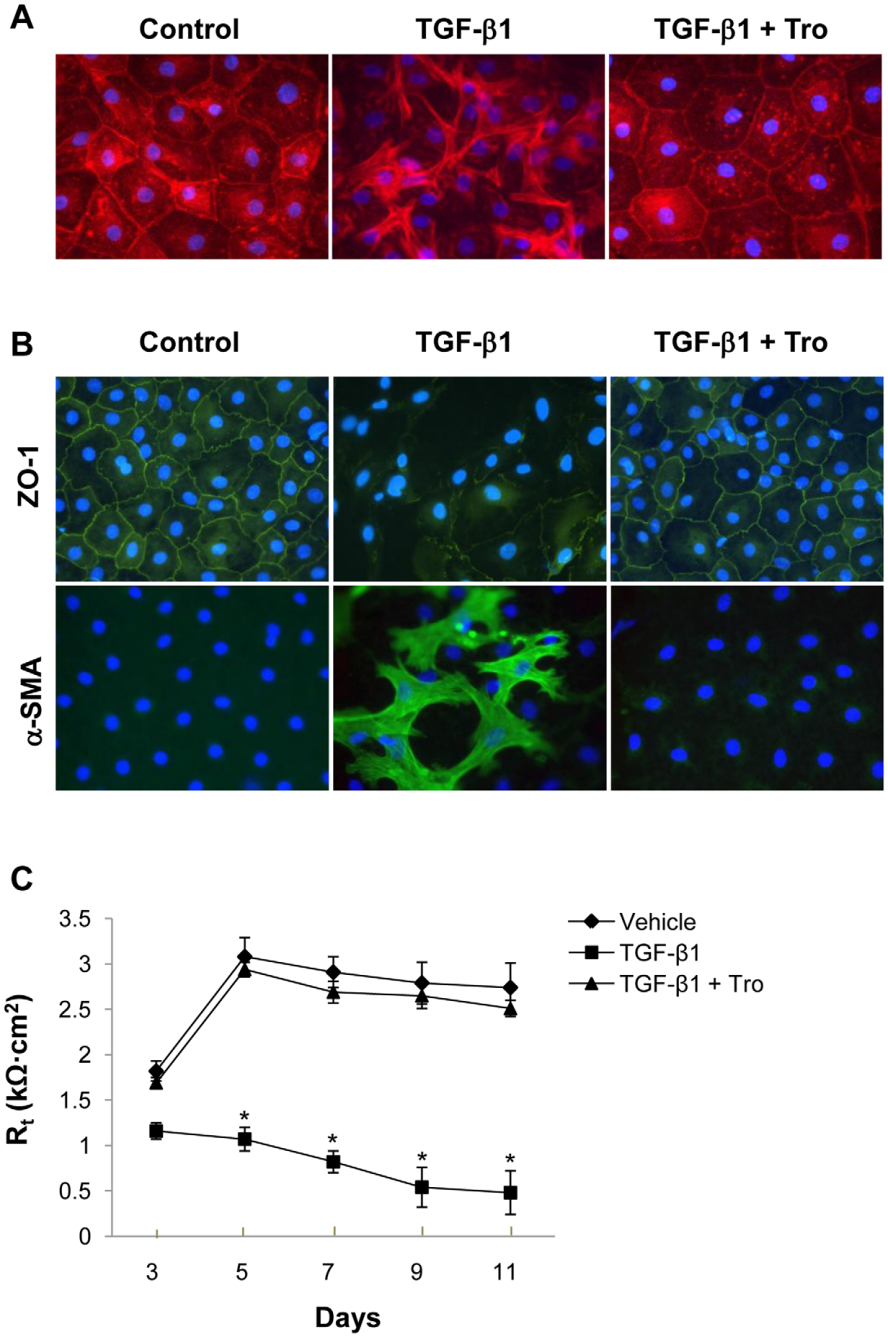
Troglitazone (Tro) inhibits EMT in primary AEC. A. Under control conditions, cells exhibit cobblestone appearance typical of epithelial morphology. Following treatment with TGF-β1, loss of cell-cell contacts and acquisition of fibroblast-like morphology are seen. Troglitazone attenuates TGF-β1-induced changes and maintains epithelial morphology. Nuclei are labeled with 4',6-diamidino-2-phenylindole (DAPI). B. Primary AEC treated with TGF-β1± troglitazone were fixed and stained for ZO-1 and α-SMA. Control cells exhibit ZO-1 staining along intercellular surfaces with minimal α-SMA expression. Treatment with TGF-β1 gives rise to loss of cell membrane-associated ZO-1 with a marked increase in α-SMA. Cells treated with both TGF-β1 and troglitazone maintain normal ZO-1 immunoreactivity with an absence of α-SMA. Nuclei are labeled with DAPI. C. TGF-β1 (present from day 2 onward) induces a decrease in transepithelial resistance (*R_t_*) of primary AEC monolayers. Decreases in *R_t_* are prevented by concurrent treatment with both TGF-β1 and troglitazone. *P<0.05 compared to vehicle; n = 3.

### Overexpression of PPARγ-DN in RLE-6TN Cells

RLE-6TN cells were seeded at a density of 40,000/well in 24-well-plates and transduced with virus expressing PPARγ-DN (LV-PPARγ-DN) or LV-control at MOI  = 2 on day 1 postseeding, followed by TGF-β (2.5 ng/ml) ± troglitazone (10 µM) treatment 16 hours after transduction. Protein was harvested for Western analysis of α-SMA and expression of FLAG-tagged PPARγ-DN after 4 days of treatment.

**Figure 2:**
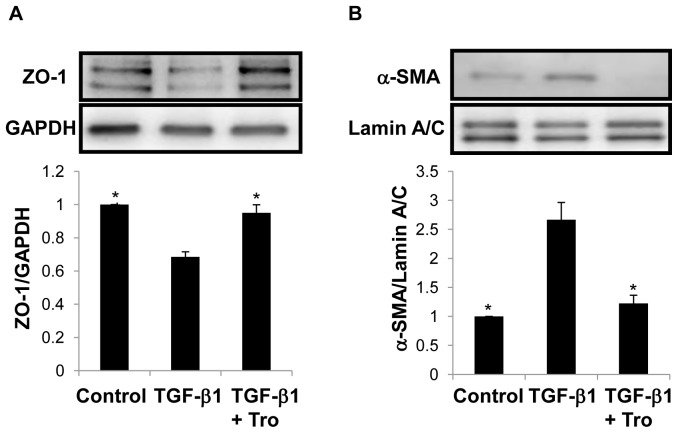
Troglitazone (Tro) prevents EMT-associated alterations in ZO-1 and α-SMA protein expression in primary AEC. Western analysis reveals inhibition of TGF-β1-mediated decreases in ZO-1 (A) and increases in α-SMA (B) by troglitazone in primary AEC. *P<0.05 compared to TGF-β1; n = 3.

### Immunofluorescence Microscopy

Rat AEC grown as monolayers on polycarbonate filters and RLE-6TN cells grown on chamber slides were fixed in 4% paraformaldehyde for 15 min and blocked in CAS Block (Invitrogen) for 1 h at RT. Filters and slides were incubated with primary antibodies overnight at 4°C and incubated with Alexa Fluor 488 conjugated secondary antibodies (Invitrogen) at RT for up to 2 h. Slides were mounted using Vectashield antifade mounting medium with 4′,6-diamidino-2-phenylindole (DAPI) or propidium iodide (PI) (Vector, Burlingame, CA) for nuclear staining. Slides were viewed with an Olympus BX60 microscope equipped with epifluorescence optics (Olympus, Melville, NY).

**Figure 3:**
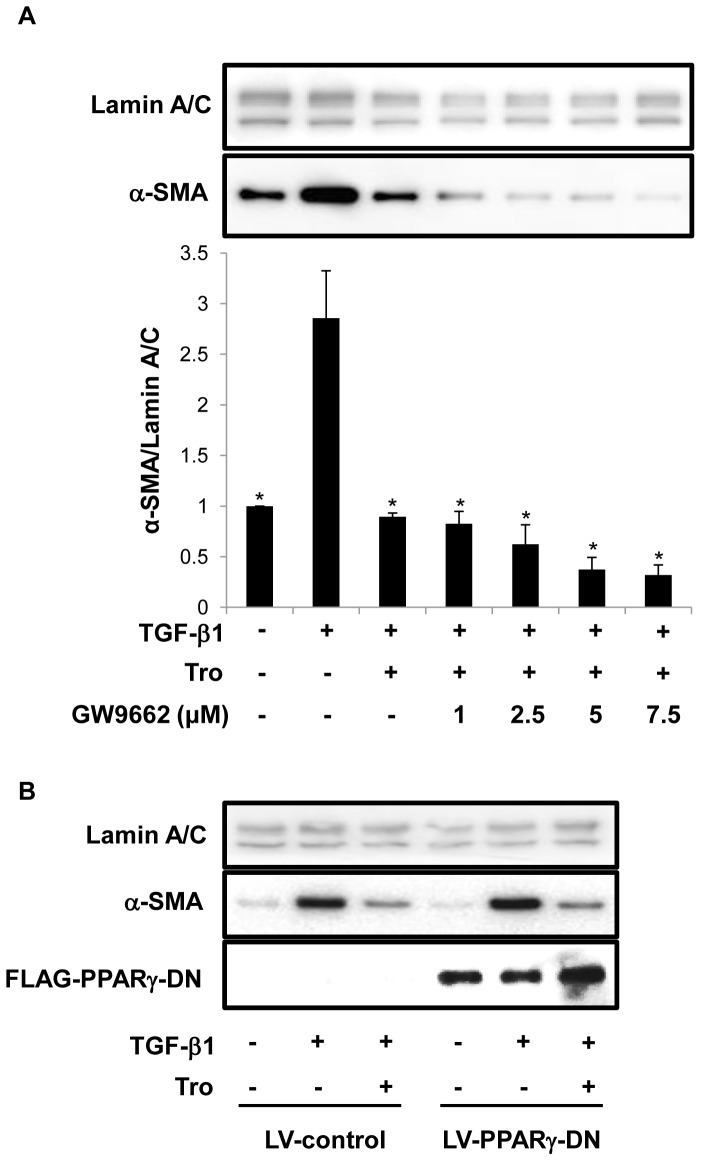
Inhibition by troglitazone (Tro) of TGF-β1-induced α-SMA expression is independent of PPARγ. A. Primary AEC were incubated with TGF-β1 (2.5 ng/ml) alone or in combination with troglitazone (10 µM) ± GW9662 (1.0–7.5 µM). Western analysis demonstrates that troglitazone prevents TGF-β1-induced increases in the myofibroblast marker α-SMA independent of PPARγ. *P<0.05 compared to TGF-β1; n = 3. B. RLE-6TN cells were transduced with virus expressing LV-PPARγ-DN or LV-control followed by TGF-β (2.5 ng/ml) ± troglitazone (10 µM) treatment for 4 days. Western analysis shows that overexpression of PPARγ-DN did not block troglitazone-mediated inhibition of α-SMA induced by TGF-β. Lamin A/C is the loading control. Data shown are representative of three separate experiments.

**Figure 4:**
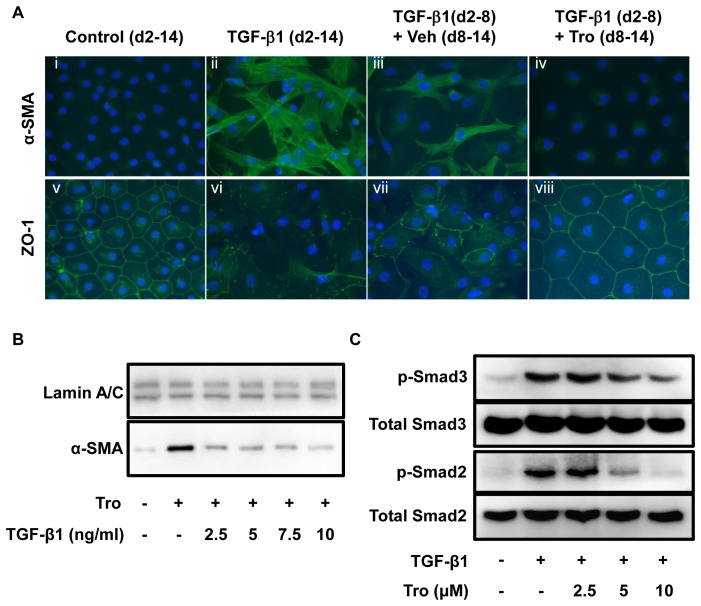
Troglitazone (Tro) reverses TGF-β1-induced EMT in primary AEC. A. Following treatment with TGF-β1 starting on day 2 for 6 days, ZO-1 immunoreactivity was markedly decreased while α-SMA was robustly expressed, reflecting that cells are undergoing EMT (*ii,vi*). Following subsequent treatment with troglitazone for an additional 6 days (from day 8 through day 14), ZO-1 expression was restored and α-SMA returned to control levels (*iv, viii*). Nuclei are labeled with DAPI. Cells treated with TGF-β1 vehicle (*i,v*) serve as negative control. TGF-β1 removal (*iii, vi*) only shows partial reversal of EMT. B. Treatment with increasing amounts of TGF-β1 (2.5–10 ng/ml) in the presence of troglitazone (10 µM) does not prevent inhibitory effects of troglitazone on TGF-β1-induced α-SMA expression. These data are representative of three separate experiments. C. Treatment with increasing amounts of troglitazone (2.5–10 µM) in the presence of TGF-β1 (2.5 ng/ml) for 2 hours reduced phosphorylation of Smad2 and Smad3 induced by TGF-β1. These data are representative of two separate experiments.

### Statistics

Data are shown as mean ± SE (standard error of the mean). Significance (*P*<0.05) for more than or equal to 3 group means was determined by one-way analysis of variance followed by post hoc procedures based on Student-Newman-Keuls approaches. Where applicable, two group means were compared for significance using Student's *t*-tests. Z-tests were used to determine if ratiometric data (i.e., normalized) were different from control.

**Figure 5:**
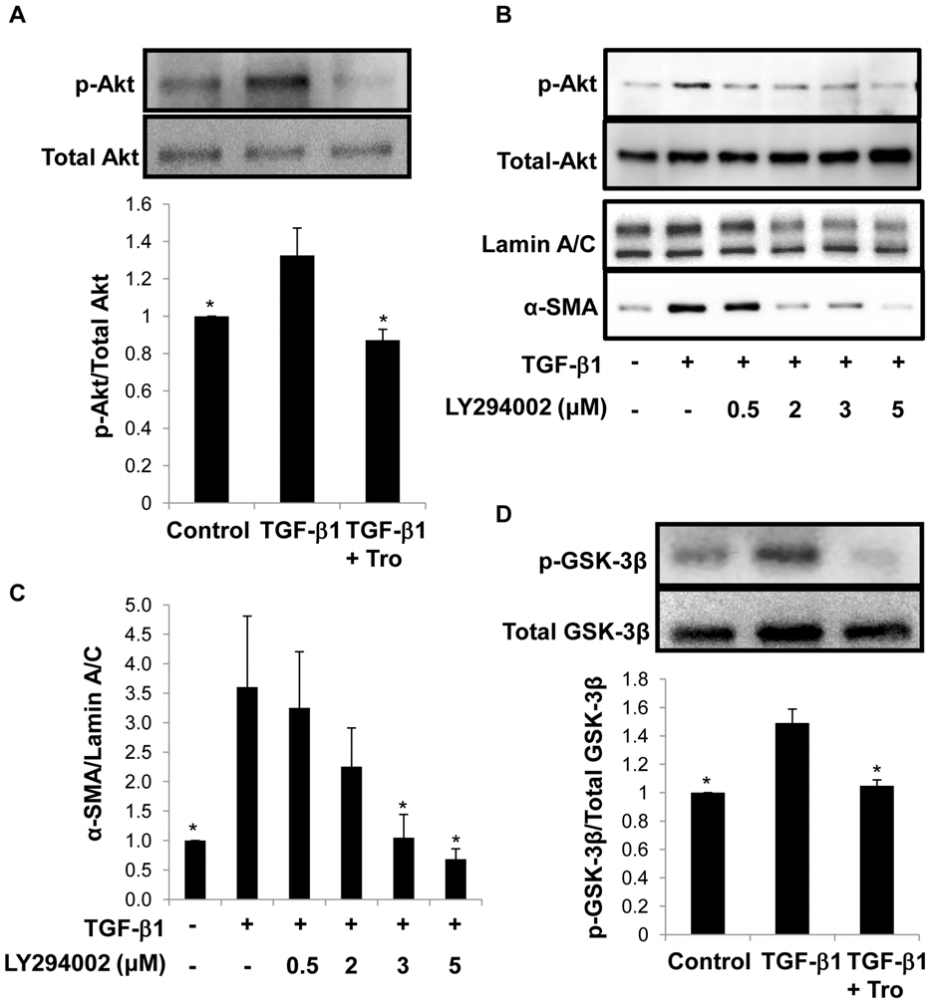
Troglitazone (Tro) inhibits TGF-β1-mediated phosphorylation of Akt and GSK-3β in primary AEC. A. Following treatment with TGF-β1 for 1 h, primary AEC exhibit marked phosphorylation of Akt at Ser437 by Western analysis. Concomitant treatment with troglitazone (10 µM) and TGF-β1 (2.5 ng/mL) attenuated Akt phosphorylation. Membranes used for Western analysis were stripped and re-probed for total Akt to confirm equal protein loading and for normalization of p-Akt levels. *P<0.05 compared to TGF-β1; n = 3). B. Concomitant treatment with the PI3-K/Akt inhibitor LY294002 (0.5–3 µM) and TGF-β1 (2.5 ng/ml) attenuated Akt phosphorylation and subsequent induction of α-SMA by TGF-β1 in primary AEC. C. Quantitative analysis of α-SMA protein in primary AEC concomitantly treated with LY294002 (0.5–3 µM) and TGF-β1. *P<0.05 compared to TGF-β1; n≥3. D. Following treatment with TGF-β1 for 2 h, primary AEC exhibit marked phosphorylation of GSK-3β by Western analysis. Concomitant treatment with troglitazone (10 µM) and TGF-β1 (2.5 ng/mL) attenuated GSK-3β phosphorylation. Membranes were re-probed for total GSK-3β to confirm equal protein loading and for normalization of pGSK-3β levels. *P<0.05 compared to TGF-β1; n = 3.

## Results

### Troglitazone Inhibits TGF-β1-induced EMT in AEC

To evaluate the influence of troglitazone on TGF-β1-induced EMT, cell morphology and expression of relevant epithelial and mesenchymal markers were evaluated. Phalloidin, which binds to filamentous actin (F-actin), was used to assess cell morphology. Following treatment with TGF-β1 for 12 days, primary AEC exhibited a marked alteration in cell morphology, changing from the characteristic organized ‘cobblestone’ appearance of differentiated epithelial cell monolayers to a disorganized elongated fibroblast-like phenotype (Figure 1A). Cells treated with 10 µM troglitazone in the presence of TGF-β1 maintained their cobblestone shape, consistent with conservation of epithelial phenotype. Similar morphological changes were noted in RLE-6TN cells (Figure S1).

**Figure 6:**
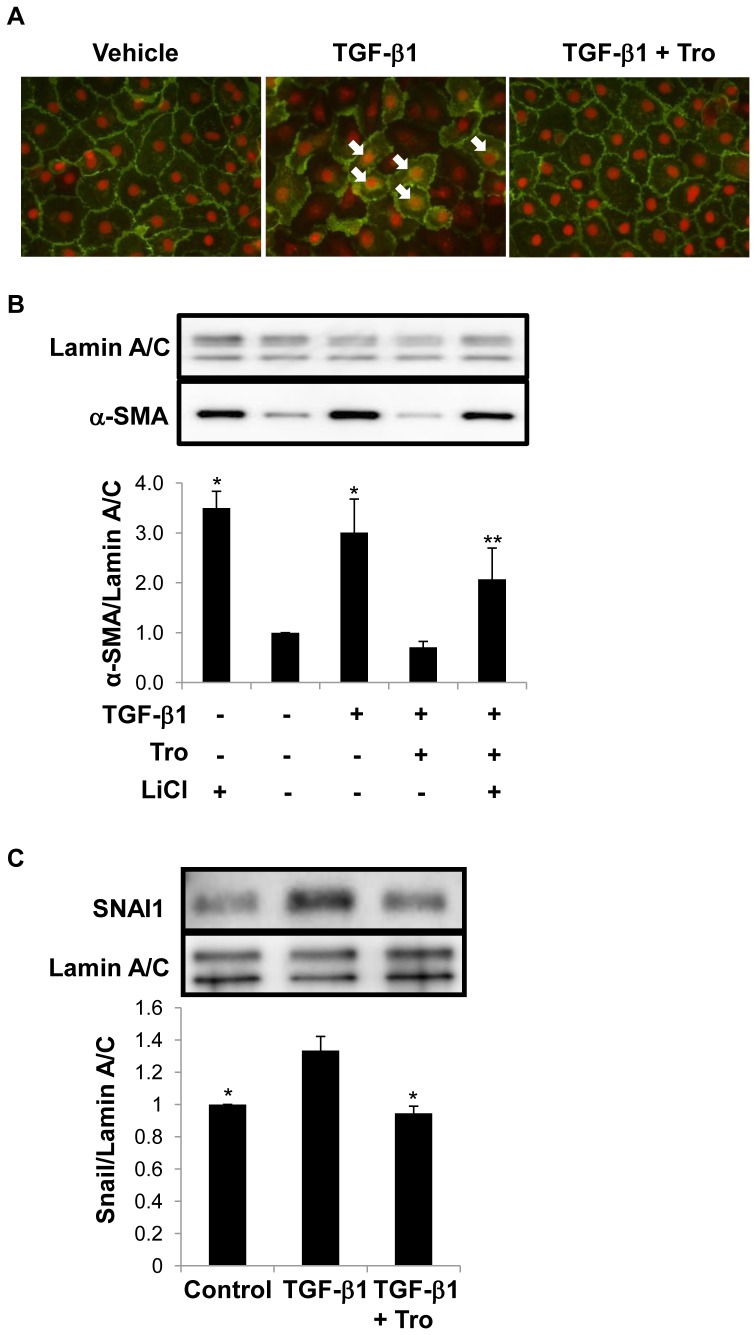
Troglitazone (Tro) abrogates TGF-β1-induced β-catenin nuclear translocation and SNAI1 expression in primary AEC. A. Membrane localization of β-catenin decreased while nuclear/perinuclear β-catenin (white arrows) increased in TGF-β1-treated cells compared to untreated (vehicle) controls. Concurrent treatment with both TGF-β1 and troglitazone maintained β-catenin at the cell plasma membrane and prevented β-catenin nuclear translocation. Nuclei are labeled with propidium iodide. *P<0.05 compared to TGF-β1; n = 3. B. Following concomitant treatment with LiCl (7.5 mM), troglitazone and TGF-β1 (2.5 ng/ml), LiCl prevented inhibition of TGF-β1-mediated α-SMA expression by troglitazone. *P<0.05 compared to vehicle; **P<0.05 compared to vehicle in the presence of TGF-β1 and troglitazone; n = 4. C. SNAI1 activity was increased upon stimulation with TGF-β1 and attenuated with troglitazone by Western analysis. *P<0.05 compared to TGF-β1; n = 3.

To assess changes in epithelial and mesenchymal markers, we investigated expression of ZO-1 (as a measure of junctional integrity and epithelial organization) and α-SMA (a characteristic mesenchymal marker). Following treatment with TGF-β1, primary AEC exhibited marked downregulation of ZO-1 relative to cells under control conditions, and expression of α-SMA dramatically increased (Figure 1B, middle panel). Importantly, in primary AEC, simultaneous treatment with both troglitazone and TGF-β1 led to maintenance of ZO-1 reactivity along cell borders with no increase in α-SMA (Figure 1B, right panel). Moreover, the integrity of AEC monolayers was maintained as indicated by preservation of *R_t_* (Figure 1C). Similarly, RLE-6TN cells exhibited a marked increase in expression of α-SMA and a decrease in expression of ZO-1 following TGF-β1 stimulation (Figure S2). These effects of TGF-β1 were inhibited by troglitazone treatment.

Consistent with immunofluorescence findings, Western analysis of primary AEC revealed diminished levels of ZO-1 and increased α-SMA expression following treatment with TGF-β1 (Figures 2A and 2B). In cells treated with troglitazone and TGF-β1, expression of both ZO-1 and α-SMA were unchanged compared to control cells treated with vehicle for both conditions (Figures 2A and 2B). Furthermore, in RLE-6TN cells, inhibition by troglitazone of TGF-β1-induced increase in α-SMA was found to be dose-dependent (Figure S3), with evidence of toxicity at 20 µM. To test whether effects of troglitazone were specific to this agent or a more generic effect of PPARγ ligands, we tested effects of two other troglitazone analogues, rosiglitazone and CAY10410, on α-SMA activation by TGF-β. Rosiglitazone inhibited TGF-β-induced α-SMA expression in RLE-6TN cells (Figure S4), but CAY10410 (which lacks an electrophilic center) did not show any inhibitory effect (data not shown). These data suggest that inhibitory effects of PPARγ ligands on EMT are dependent on their physical properties, similar to a previous report in the context of fibroblast-myofibroblast differentiation [23].

### Inhibitory Effects of Troglitazone are Independent of PPARγ

Consistent with previous studies showing that PPARγ is widely expressed in lung, including in AEC [24], [25], RNA profile analysis (Illumina RatRef-12) using freshly isolated AT2 cells from rat lung and AT1-like cells cultivated *in vitro* for 8 days confirmed expression of PPARγ (data not shown). In order to determine if troglitazone exerts its inhibitory effects via PPARγ-dependent or -independent pathways, primary AEC were concurrently treated with troglitazone and TGF-β1 in the presence or absence of GW9662, a selective irreversible antagonist of PPARγ. As shown by Western analysis (Figure 3A), troglitazone inhibited TGF-β1-mediated increases in α-SMA expression in primary AEC. However, blockade of PPARγ using GW9662 (1.0 to 7.5 µM) failed to antagonize inhibitory actions of troglitazone (Figure 3A). To further confirm that PPARγ is not involved in troglitazone-mediated inhibition, RLE-6TN cells were transduced with lentivirus expressing a PPARγ dominant negative construct (LV-PPARγ-DN) or control (LV-control), followed by treatment with TGF-β and/or troglitazone. Overexpression of a LV-PPARγ-DN did not prevent troglitazone-mediated inhibition of α-SMA induction by TGF-β (Figure 3B), indicating that attenuation of EMT by troglitazone is primarily mediated by PPARγ-independent pathway(s).

### Troglitazone Reverses TGF-β1-induced EMT

While several pharmacological agents have been shown to inhibit EMT, few exhibit the ability to also reverse this process. Accordingly, we assessed troglitazone’s capacity to reverse the characteristic alterations associated with alveolar EMT. Following acquisition of mesenchymal phenotype after stimulation with TGF-β1 for 6 days, primary AEC were treated with troglitazone. This gave rise to complete reversal of EMT-associated morphological changes, together with complete restoration of ZO-1 at cell borders and return of α-SMA expression to control levels, when assessed 6 days after onset of troglitazone treatment (Figure 4A, fourth panel). In contrast, simple removal of TGF-β1 led to only partial reversion of EMT by day 14 (Figure 4A, third panel). To further examine whether troglitazone works as a competitive inhibitor of TGF-β1 binding to the TGF-β1 receptor, primary cells were treated with troglitazone (10 µM) and increasing concentrations of TGF-β (2.5, 5, 7.5 and 10 ng/ml). As shown in Figure 4B, increasing concentrations of TGF-β did not overcome inhibitory effects of troglitazone. Nevertheless, troglitazone inhibited phosphorylation of both Smad3 and Smad2 in a dose-dependent manner, suggesting that TGF-β-mediated EMT is Smad-dependent and that troglitazone effects involve signaling via TGF-β receptors (Figure 4C).

### Troglitazone Inhibits TGF-β1-associated Phosphorylation of Akt and GSK-3β

TGF-β1-induced EMT is associated with activation of numerous intracellular signaling pathways. We found that TGF-β1 induced phosphorylation of Akt at Ser437 in primary AEC (Figure 5A). When cells were treated concomitantly with troglitazone and TGF-β1, activation of Akt was inhibited (Figure 5A), indicating that troglitazone modulates Akt phosphorylation. Furthermore, treatment with the PI3-K/Akt pathway specific inhibitor LY294002 showed inhibition of TGF-β-induced Akt phosphorylation and subsequent α-SMA induction in a dose-dependent manner (Figure 5B and 5C), confirming a role for signaling via PI3-K/Akt in TGF-β1-induced EMT.

Having established troglitazone’s ability to inhibit TGF-β1-induced phosphorylation of Akt, we explored potential signaling pathways downstream of Akt. Akt phosphorylates a variety of substrates, including GSK-3β [26]. Inhibition of GSK-3β activity by phosphorylation mediates disruption of epithelial junctional complexes coupled with nuclear translocation of β-catenin (an important component of EMT) [27]. TGF-β1 increased levels of pGSK-3β relative to total GSK-3β (Figure 5D). However, concomitant treatment with troglitazone blocked this process such that GSK-3β activity was maintained at levels comparable to that of controls (Figure 5D).

### Inhibition of TGF-β1-induced Nuclear Translocation of β-catenin and SNAI1 Activation by Troglitazone

When stimulated with TGF-β1, AEC exhibited marked accumulation of β-catenin in nuclear and peri-nuclear regions, as shown by immunofluorescence (Figure 6A), which was markedly reduced following simultaneous treatment with troglitazone (Figure 6A). To further test the importance of nuclear accumulation of β-catenin, we treated cells with a combination of TGF-β, troglitazone and LiCl (an activator of the Wnt pathway by inactivation of GSK-3β) [28]. As shown in Figure 6B, treatment with LiCl prevented troglitazone-mediated inhibition of α-SMA by TGF-β, suggesting that troglitazone effects are mediated, at least in part, by inhibition of TGF-β-induced nuclear accumulation of β-catenin. Similarly, TGF-β1 was shown to upregulate SNAI1 in AEC, as shown by Western analysis (Figure 6C). Moreover, concurrent treatment with troglitazone effectively inhibited EMT-related stabilization of SNAI1 (Figure 6C). Taken together, these results suggest that troglitazone inhibits EMT via an Akt- and GSK-3β-dependent pathway, effecting changes in β-catenin- and SNAI1-related signaling.

## Discussion

Evidence continues to accumulate indicating that natural and synthetic PPARγ ligands exert beneficial effects in experimental models of IPF [14], [15]. Mechanisms by which PPAR ligands exert their antifibrogenic effects are poorly understood but potentially involve numerous complementary pathways, including antagonism of TGF-β signaling, upregulation of phosphatase and tensin homologue deleted on chromosome 10 (PTEN) and increased hepatocyte growth factor activity [29]. Specifically, PPARγ ligands have been shown to attenuate TGF-β1-driven differentiation of both pulmonary- and hepatic-derived fibroblasts to myofibroblasts [9]. EMT has been shown to contribute to myofibroblast accumulation in the lung *in vivo* and is primarily driven by TGF-β1 [5]. For these reasons, EMT and its underlying mechanisms represent attractive targets for pharmacological intervention in IPF. In the current study, we investigated a potential therapeutic approach for maintenance and restoration of alveolar epithelial integrity via inhibition of TGF-β1-induced EMT with troglitazone. We demonstrate that, in both primary rat AEC and RLE-6TN cells, troglitazone maintained epithelial morphology and cell-cell junctional architecture when cells were challenged with TGF-β1. Moreover, troglitazone blocked TGF-β1-mediated changes in ZO-1 distribution and increases in α-SMA expression, consistent with inhibition of EMT.

Although inhibition of EMT offers the possibility of slowing or halting the fibrogenic process, existing EMT-associated fibrotic lesions could remain unaffected. Thus, from a therapeutic perspective, reversal of both EMT and fibrosis is especially desirable. In addition to troglitazone’s strongly antifibrotic activity and its observed inhibition of EMT, our results show that troglitazone is able to revert established α-SMA-expressing (myo-) fibroblasts to their original epithelial phenotype. Troglitazone may therefore represent a promising therapeutic agent with which to effectively facilitate re-epithelialization within the lung.

It is known that TZDs and other agonists such as 15d-PGJ2 exhibit both PPARγ-dependent and -independent effects [30]. Several lung-related studies emphasizing the anti-fibrotic role of these agents have indicated PPARγ-independent effects [9], [11], [15], although these questions have not been addressed in the context of EMT in primary AEC. In order to explore if this inhibition of TGF-β1-induced EMT is PPARγ-dependent, we employed an irreversible PPARγ antagonist GW9662 in combination with a PPARγ DN approach (Figure 3) to show that troglitazone’s effect is independent of PPARγ. Interestingly, it has been demonstrated that both GW9662 (whose actions are mediated predominantly via PPARγ) and PPARγ DN are poor inhibitors of fibroblast-to-myofibroblast differentiation [23]. Mechanisms underlying PPARγ-independent effects of these agonists have not been fully characterized, especially in the context of EMT. A recent study revealed that both rosiglitazone and cioglitazone effectively inhibit key components of EMT in the A549 alveolar adenocarcinoma cell line via a Smad-independent mechanism [18]. In contrast, work by Reka and colleagues [19] suggested that troglitazone and rosiglitazone antagonize Smad3 signaling during TGF-β1-induced EMT in A549 cells in a PPARγ-dependent fashion, leaving the precise mechanism(s) unresolved, although mechanistic observations derived from cell lines of cancerous origin may not be effectively translated to the *in vivo* setting in the context of IPF.

To further address mechanisms by which troglitazone inhibits EMT in non-malignant AEC, we focused on components downstream of TGF-β1 signaling. Activation of the Akt pathway in response to TGF-β1 has been shown to mediate EMT in non-malignant mammary and renal epithelial cells [31], [32]. Moreover, inhibition of Akt activity attenuated TGF-β1-mediated EMT in rat kidney epithelial cells [33], while in oral squamous cell carcinoma, Akt inhibition induces mesenchymal-to-epithelial transition [34]. Our findings indicate that troglitazone inhibits TGF-β1-mediated phosphorylation of Akt at Ser437, while the PI3-K inhibitor LY294002 inhibits Akt phosphorylation and α-SMA induction in response to TGF-β1 (Figure 5), suggesting a novel pathway by which troglitazone attenuates EMT of AEC, consistent with observations in other cellular systems [35]–[37].

Inactivation of GSK-3β, a key downstream effector of Akt, leads to stabilization of SNAI1 and β-catenin, both key mediators of EMT [26], [27], [38]–[40]. We recently reported that TGF-β-induced phosphorylation of β-catenin at Tyr654 and dephosphorylation at Ser37 and Thr41, in conjunction with interaction of β-catenin with Smad3 and CBP, upregulates α-SMA expression during TGFβ1-induced EMT in AEC [41]. This led us to postulate that troglitazone’s inhibitory effects on TGFβ1-mediated EMT may be mediated by inhibition of both β-catenin- and SNAI-dependent signaling downstream of the PI3-K/Akt/GSK-3β pathway. Consistent with this hypothesis, we demonstrate significant reductions in SNAI1 expression (Figure 6C), and inhibition of nuclear translocation of β-catenin (Figure 6A), upon concurrent treatment with troglitazone and TGF-β1. Although PPARγ ligands are known to inhibit β-catenin signaling [42], this is the first demonstration to our knowledge that TZDs oppose effects of TGF-β on EMT by modulating β-catenin and SNAI1 activation via PI3-K/Akt/GSK-3β signaling. Consistent with our findings, a recent study in renal proximal tubular cells showed an inhibitory effect of troglitazone on SNAI1 expression and β-catenin nuclear translocation in EMT induced by high glucose [17]. In addition to troglitazone’s inhibition of TGF-β1 action, PPARγ ligands have also been shown to reduce TGF-β1 synthesis, both *in vivo* [15] and *in vitro* [43]. While our findings have revealed a novel molecular pathway by which troglitazone overrides profibrotic action of TGF-β1, effects on TGF- β1 synthesis by AEC remain to be elucidated.

The present study reveals effectiveness of troglitazone in attenuation of TGF-β-induced EMT in AEC by inhibiting a PI3-K/Akt- and GSK-3β-dependent pathway responsible for key EMT events, namely, SNAI1 upregulation and β-catenin activation. Our data suggest a potentially useful role for troglitazone as a therapeutic agent to reduce and/or reverse EMT of alveolar epithelium associated with IPF, in which colocalization of β-catenin and Smad3 have been identified in hyperplastic AT2 cells [41]. Although systemically administered troglitazone has been shown to exhibit hepatotoxic effects in some instances [44], employment of aerosol therapy could facilitate a reduction in the rate and severity of any potential off-target effects, as have been shown for other drugs (e.g., inhaled corticosteroids and beta-agonists). Alternatively, since rosiglitazone similarly inhibits TGF-β effects, our results suggest that effects of troglitazone on EMT may be generalizable to the TZD subclass of PPARγ ligands.

## Supporting Information

Figure S1 **Troglitazone (Tro) attenuates TGF-β1-induced changes in morphology of RLE-6TN cells.** Under control conditions, cells exhibit cobblestone appearance typical of epithelial morphology. Following treatment with TGF-β1, loss of cell-cell contacts and acquisition of fibroblast-like morphology are seen. Troglitazone attenuates TGF-β1-induced changes and maintains epithelial morphology. Nuclei are labeled with 4′,6-diamidino-2-phenylindole (DAPI). Data are representative of two separate experiments.
(TIF)

Figure S2 **Troglitazone (Tro) inhibits EMT in RLE-6TN cells.** Following treatment with TGF-β1± troglitazone for 3 days, RLE-6TN cells were fixed and stained for ZO-1 and α-SMA. Control cells exhibit ZO-1 staining along intercellular surfaces with minimal α-SMA. Treatment with TGF-β1 gives rise to loss of membrane-associated ZO-1 with a marked increase in α-SMA expression. Cells treated concurrently with both TGF-β1 and troglitazone maintain ZO-1 immunoreactivity and absence of α-SMA. Nuclei are labeled with 4′,6-diamidino-2-phenylindole (DAPI). Data are representative of three separate experiments.
(TIF)

Figure S3 **Effects of troglitazone (Tro) on α-SMA expression are dose dependent.** RLE-6TN cells were treated with TGF- β1 in the presence of increasing doses of troglitazone. Representative Western blot demonstrates dose-dependent reduction in α-SMA.
(TIF)

Figure S4 **Effects of rosiglitazone (Ros) on α-SMA expression are dose dependent.** RLE-6TN cells were treated with TGF-β1 in the presence of increasing doses of rosiglitazone. Representative Western blot (upper panel) and quantitation (lower panel) demonstrate dose-dependent reductions in α-SMA induced by TGF-β1. *P<0.05 compared to TGF-β1; n = 3. GAPDH is used as loading control.
(TIF)
