# Supplementary figures and images for: Troglitazone Attenuates TGF-β1-Induced EMT in Alveolar Epithelial Cells via a PPARγ-Independent Mechanism
Source: PLoS One. 2012 Jun 20;7(6):e38827. doi: 10.1371/journal.pone.0038827 (PMC3380041; doi:10.1371/journal.pone.0038827)

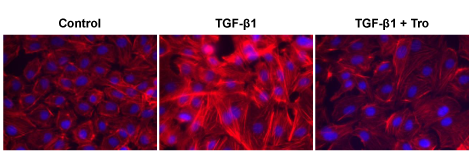

Supplement: Figure S1 — Troglitazone (Tro) attenuates TGF-β1-induced changes in morphology of RLE-6TN cells. Under control conditions, cells exhibit cobblestone appearance typical of epithelial morphology. Following treatment with TGF-β1, loss of cell-cell contacts and acquisition of fibroblast-like morphology are seen. Troglitazone attenuates TGF-β1-induced changes and maintains epithelial morphology. Nuclei are labeled with 4′,6-diamidino-2-phenylindole (DAPI). Data are representative of two separate experiments. (TIF) [file pone.0038827.s001.tif]

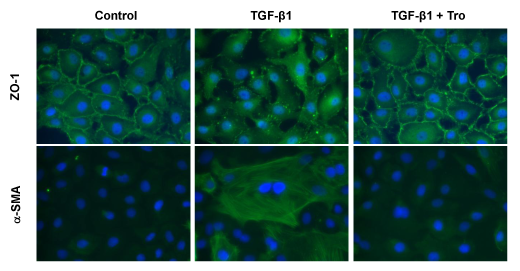

Supplement: Figure S2 — Troglitazone (Tro) inhibits EMT in RLE-6TN cells. Following treatment with TGF-β1± troglitazone for 3 days, RLE-6TN cells were fixed and stained for ZO-1 and α-SMA. Control cells exhibit ZO-1 staining along intercellular surfaces with minimal α-SMA. Treatment with TGF-β1 gives rise to loss of membrane-associated ZO-1 with a marked increase in α-SMA expression. Cells treated concurrently with both TGF-β1 and troglitazone maintain ZO-1 immunoreactivity and absence of α-SMA. Nuclei are labeled with 4′,6-diamidino-2-phenylindole (DAPI). Data are representative of three separate experiments. (TIF) [file pone.0038827.s002.tif]

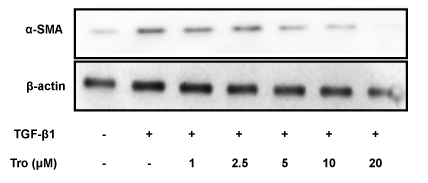

Supplement: Figure S3 — Effects of troglitazone (Tro) on α-SMA expression are dose dependent. RLE-6TN cells were treated with TGF- β1 in the presence of increasing doses of troglitazone. Representative Western blot demonstrates dose-dependent reduction in α-SMA. (TIF) [file pone.0038827.s003.tif]

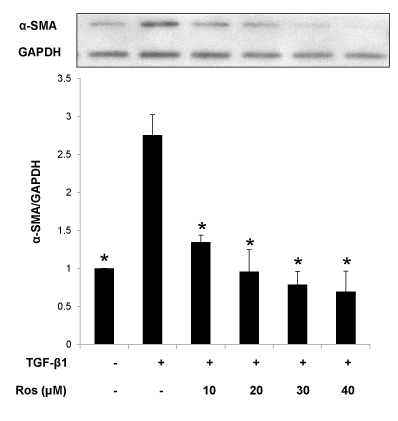

Supplement: Figure S4 — Effects of rosiglitazone (Ros) on α-SMA expression are dose dependent. RLE-6TN cells were treated with TGF-β1 in the presence of increasing doses of rosiglitazone. Representative Western blot (upper panel) and quantitation (lower panel) demonstrate dose-dependent reductions in α-SMA induced by TGF-β1. *P<0.05 compared to TGF-β1; n = 3. GAPDH is used as loading control. (TIF) [file pone.0038827.s004.tif]
